# A Multimodal Diagnostic Algorithm for Focal Knee Chondral Defects: Correlating Clinical Tests, Musculoskeletal Ultrasound, and MRI-Based ICRS Grading

**DOI:** 10.3390/life16010080

**Published:** 2026-01-05

**Authors:** Robert Gherghel, Paul-Dan Sîrbu, Elena Rezus, Sonia Gabriela Neagu, Carmina Liana Musat, Georgiana Bianca Constantin, Daniel Madalin Coja, Corneliu Mircea Codreanu, Daniel Andrei Iordan, Ilie Onu

**Affiliations:** 1 Department of Orthopaedic and Traumatology, Grigore T. Popa University of Medicine and Pharmacy Iasi, 700115 Iasi, Romania; gherghelrobert@yahoo.com (R.G.); paul.sirbu@umfiasi.ro (P.-D.S.); 2 Department of Rheumatology and Rehabilitation, Grigore T. Popa University of Medicine and Pharmacy Iasi, 700115 Iasi, Romania; elena.rezus@umfiasi.ro; 3 Departments of Orthopedy and Physiotherapy, Medlife-Micromedica Clinic, 610119 Piatra Neamt, Romania; 4 Department of Environment Science, Physics, Physical Education and Sport, Faculty of Science, University “Lucian Blaga”, 550012 Sibiu, Romania; 5 Faculty of Medicine and Pharmacy, “Dunarea de Jos” University of Galati, 800008 Galati, Romania; carmina.musat@ugal.ro (C.L.M.); bianca.constantin@ugal.ro (G.B.C.); 6 Center of Physical Therapy, Rehabilitation and Wellness, “Dunărea de Jos” University of Galati, 800008 Galati, Romania; daniel.coja@ugal.ro (D.M.C.); corneliu.codreanu@ugal.ro (C.M.C.); ilie.onu@umfiasi.ro (I.O.); 7 Department of Individual Sports and Kinetotherapy, Faculty of Physical Education and Sport, “Dunarea de Jos” University of Galati, 800008 Galati, Romania; 8 Department of Biomedical Sciences, Grigore T. Popa University of Medicine and Pharmacy Iasi, 700454 Iasi, Romania

**Keywords:** focal chondral lesions, ICRS grading, knee cartilage injury, musculoskeletal ultrasound (MSK-US), MRI cartilage assessment

## Abstract

Background: Focal chondral lesions of the knee are frequently underdiagnosed, and their clinical presentation does not always correlate with structural severity. This study aimed to evaluate the diagnostic utility of clinical examination tests and musculoskeletal ultrasound (MSK-US) in identifying high-grade chondral defects, using MRI-based ICRS grading as the reference standard. Methods: In this observational cross-sectional study, 57 consecutive patients with mechanical knee pain and MRI-confirmed focal chondral lesions were evaluated through standardized clinical examination, MSK-US, and MRI. Clinical maneuvers—including Wilson’s test, McMurray’s test, and ligamentous stability tests—were analyzed using Chi-square tests, Pearson correlations, and odds ratios (OR). Statistical processing was performed in Python. Results: According to MRI grading, 87.7% of lesions were ICRS 3, and 12.3% were ICRS 4. Pain and functional impairment (as measured by the WOMAC) were moderate and comparable across lesion grades. Wilson’s test showed high sensitivity in both ICRS 3 (66%) and ICRS 4 (100%) lesions, but no statistical association with lesion severity (*p* = 0.955). McMurray’s test demonstrated strong discriminative value, being positive in 30% of ICRS 3 versus 86% of ICRS 4 lesions, and was the only clinical maneuver significantly associated with lesion grade (χ^2^ = 4.29, *p* = 0.038; OR = 0.20, 95% CI: 0.05–0.79). Correlation analysis revealed weak associations between clinical tests and the location of compartment-specific defects. MRI identified meniscal tears in 86% of ICRS 4 lesions compared with 30% of ICRS 3 lesions. Conclusions: Symptom severity alone does not reliably distinguish between ICRS Grade 3 and Grade 4 focal chondral lesions. McMurray’s test, while not cartilage-specific, was associated with lesion complexity due to its reflection of concomitant meniscal pathology rather than cartilage depth itself. Accordingly, McMurray’s test should be interpreted as an indirect clinical indicator of combined osteochondral–meniscal involvement. The integration of targeted clinical tests (Wilson’s and McMurray’s), MSK-US and MRI-based ICRS grading may support clinical orientation and preoperative risk stratification, forming a pragmatic diagnostic framework rather than a definitive staging tool.

## 1. Introduction

Chondropathy represents the progressive degradation of joint cartilage, a tissue with extremely limited self-healing potential due to its avascular, aneural, and alymphatic structure. Even small, untreated lesions can evolve into severe osteoarthritis (OA), as spontaneous recovery is rare and current medical technologies cannot fully restore cartilage integrity [1,2].

Chondral lesions may result from metabolic, genetic, vascular, or traumatic causes, appearing after either a single excessive load or repetitive minor stress on the joint. Depending on the depth of damage, they are classified as partial-thickness or full-thickness (osteochondral) defects involving both cartilage and subchondral bone. Although some remain asymptomatic, these lesions frequently progress to knee OA (KOA) if untreated [3,4].

Macroscopic cartilage damage is commonly graded using the Outerbridge classification (grades 1–4), while the International Cartilage Repair Society (ICRS) system is preferred in studies evaluating cartilage repair and regeneration [5,6,7].

The true incidence of osteochondral lesions in humans is difficult to determine, as many cases remain asymptomatic. Retrospective studies, however, provide valuable insights into their prevalence. Song and Park estimated that approximately 12% of the population presents cartilage damage in the knee [8]. Given the current trend of increasing participation in recreational sports, the prevalence of such injuries is expected to rise further. Cartilage lesions have been identified in up to 66% of all knee arthroscopies, highlighting their high occurrence and potential progression to KOA [1,8].

Curl et al. reported that 41% of patients with chondral lesions observed during arthroscopy had fragmentation and fissuring over areas larger than 1.5 cm^2^ (Outerbridge grade III). In comparison, 19.2% showed erosion extending to the subchondral bone (grade IV). Among grade IV lesions, 20% were located in the medial femoral condyle, and 72% occurred in patients over 40 years old [9].

Widuchowski et al. found that 60% of knees undergoing surgery exhibited some form of chondral damage, of which 67% were chondral or osteochondral lesions, 29% were osteoarthritis, 2% osteochondritis dissecans, and 1% other conditions—around 70% of lesions extended beyond the cartilage layer, while 30% were confined to cartilage. The patellar surface (36%) and medial femoral condyle (34%) were the most frequent sites, with grade 2 lesions being most common (42%). These often coexisted with medial meniscus tears (42%) or anterior cruciate ligament (ACL) injuries (36%) [10].

### 1.1. Clinical and Functional Examination

The initial step in evaluating articular cartilage lesions involves obtaining a comprehensive medical history, including age (typically over 40 years) and body mass index (BMI), followed by a thorough physical examination to identify the mechanism of injury and current symptoms.

Common clinical manifestations include joint swelling, mechanical pain, limping, and a sensation of locking or instability. Before initiating treatment, it is essential to rule out other possible causes of joint pathology and to consider any previous treatments or surgical interventions [11,12].

The physical examination should assess gait pattern, joint effusion, static and dynamic knee alignment, active and passive range of motion (ROM), proximal muscle strength, and the condition of the patellofemoral joint. Evaluation of crepitus at the patellofemoral or tibiofemoral joint, as well as knee stability, is crucial. Lateral instability or positive anterior/posterior drawer signs may indicate insufficiency of the collateral or cruciate ligaments.

Joint effusion of the knee often suggests symptomatic cartilage damage, provided that other causes have been excluded. The ROM should be compared with the contralateral side, with particular attention to extension deficits, which may signal progression to KOA beyond the reparative potential of cartilage procedures. Measurements should include passive hyperextension, active extension, and passive flexion [13].

Mechanical symptoms such as catching or locking during flexion or extension may indicate the presence of an unstable meniscus, a detached cartilage fragment, or a loose intra-articular body [13]. Knee stability is a prerequisite for cartilage repair, thus requiring a complete ligamentous examination, including varus and valgus stress tests at 0° and 30°, as well as the anterior drawer and Lachman tests.

The Lachman test is used to assess the ACL and meniscal integrity. With the subject in a supine position and the knee flexed at 20–30°, the examiner stabilizes the distal thigh with one hand and grasps the tibia with the other, placing the thumb on the tibial tuberosity. An anterior force is applied to translate the tibia forward relative to the femur. A positive Lachman test, indicated by pain or excessive anterior translation, suggests an ACL rupture or possible meniscal injury [14,15].

The anterior drawer test is used in the initial clinical evaluation of a suspected ACL rupture. The subject lies in a supine position with the hips flexed at 45°, the knees flexed at 90°, and the feet flat on the examination table. The examiner sits in front of the tested knee, grasping the tibia just below the joint line. The tibia is then briskly pulled forward in an anterior direction. A marked anterior translation of the tibia compared to the contralateral limb, or the absence of a firm endpoint, may indicate either a sprain of the anteromedial bundle or a complete ACL rupture [14,15].

The J-sign refers to the inverted “J”-shaped trajectory that the patella follows from knee extension to early flexion (or vice versa) in cases of patellar maltracking. A laterally subluxated patella shifts abruptly medially when it engages with the trochlear groove of the distal femur. The presence of a positive J-sign during clinical examination is indicative of patellar maltracking and instability [16].

The varus stress test (adduction stress test) is used to evaluate the integrity of the lateral collateral ligament (LCL). The subject lies in a supine position with the knee flexed at 30°. While stabilizing the knee, the examiner applies an adduction force at the ankle. If the knee joint shows greater adduction than normal compared to the healthy limb, the test is considered positive [17,18].

The valgus stress test (abduction stress test) assesses the integrity of the medial collateral ligament (MCL). The subject lies in a supine position with the knee flexed at 30°. While stabilizing the femur, the examiner applies an abduction force to the ankle. If the knee joint shows greater abduction than normal compared to the contralateral limb, the test is considered positive [19,20,21].

The pivot shift test dynamically assesses knee stability and ACL integrity. With the subject supine and the leg relaxed in extension, the examiner applies an axial load and valgus force while internally rotating the tibia during knee flexion. In cases of ACL injury, the lateral tibial plateau subluxates anteriorly in extension and reduces suddenly between 20° and 40° of flexion, producing a palpable or audible shift [22,23,24].

The Posterior Drawer Test is used to assess the integrity of the posterior cruciate ligament (PCL). The patient is positioned in supine lying with the knee flexed to 90°, while the examiner stabilizes the tested limb by sitting on the patient’s forefoot. The examiner grasps the proximal tibia with both hands and applies a posterior translational force to the tibia. The test is considered positive when an excessive posterior displacement of the tibia relative to the femur is observed, indicating a PCL injury [25,26,27].

The Posterior Sag Test evaluates PCL integrity. With the patient supine and the hip and knee flexed to 90°, the examiner inspects the anterior margin of the medial tibial plateau. In a normal knee, this margin lies slightly anterior to the femoral condyle; in PCL injury, gravity causes a posterior “sag” of the tibia, indicating a positive test. The test can also be performed with the heel elevated, maintaining 90° hip and knee flexion [28].

Wilson’s Test is used to detect osteochondritis dissecans of the knee, particularly on the medial femoral condyle. The patient flexes the knee to 90° while the examiner applies internal rotation to the tibia. The patient then extends the knee while maintaining internal rotation. If pain occurs, the tibia is externally rotated; pain typically decreases. Relief of pain with external rotation suggests the presence of osteochondritis dissecans [29].

Clinical diagnosis is challenging because symptoms are nonspecific and may overlap with other knee pathologies. There are no dedicated clinical tests for assessing articular cartilage integrity, and differentiating between chondral and meniscal lesions during physical examination is often difficult. Patients commonly report pain, swelling, catching, and crepitus. Symptoms typically have an insidious onset, with diffuse or localized pain around the joint line or in the anterior knee region [3]. Certain provocation tests may assist in diagnosis, such as Wilson’s test, which helps identify osteochondritis dissecans of the medial femoral condyle [29].

### 1.2. Radiologic Evaluation

Radiologic assessment plays a key role in excluding other pathological conditions and associated injuries, such as degenerative changes characterized by osteophytes, cysts, or subchondral sclerosis. Standard imaging includes weight-bearing anteroposterior and lateral radiographs, which help identify tibiofemoral contact—considered a contraindication for cartilage repair procedures—and detect osteochondritis dissecans when present [30].

An axial patellofemoral radiograph at 30° flexion provides valuable information regarding patellar tracking and the morphology of the trochlea, which may represent a suitable donor site for osteochondral graft harvesting.

Radiographs are also useful for identifying full-thickness chondral lesions associated with loose bodies in the joint space—features typical of osteochondritis dissecans [30]. In addition, they can reveal osseous abnormalities such as fractures and are essential in evaluating malalignment. In such cases, long-leg standing radiographs are required to determine whether a corrective osteotomy is indicated [30].

Computed Tomography (CT) and arthro-CT share similar diagnostic indications with radiography, though arthro-CT uses intra-articular contrast to directly visualize chondral defects. With the widespread use of magnetic resonance imaging (MRI), these methods are no longer considered the gold standard for joint evaluation. Arthro-CT can effectively identify cartilage lesions—especially fissures and defects exceeding 50% of cartilage thickness—and offers higher specificity than MRI in these cases [31]. However, its use is limited by the risks associated with intra-articular contrast injection.

Both arthro-CT and MRI allow imaging-based estimation of osteochondral loss and defect depth according to ICRS criteria [32]. Despite its precision, arthro-CT has largely been replaced by MRI, which provides detailed, non-invasive imaging without procedural risks [33].

MRI is considered the reference imaging technique for evaluating cartilage lesions due to its excellent soft-tissue contrast. Conventional sequences such as T1 spin-echo, T1/T2 gradient-echo, and T2 sequences detect up to 53% of chondral defects, although superficial lesions may be missed [34]. In the absence of recent trauma, subchondral bone defects may indicate deeper grade 3–4 cartilage injuries [32].

Dedicated cartilage sequences—such as 3D T1 gradient-echo with fat saturation and fat-suppressed fast spin-echo T2—provide improved visualization of cartilage surface, thickness, volume, and subchondral bone [32]. MRI offers high-resolution images essential for accurate diagnosis and for evaluating associated meniscal pathology, which should be preserved whenever possible [35].

MRI and arthroscopy together help characterize the defect’s location, size, depth, and surrounding tissue quality, all critical for selecting appropriate surgical treatment [36,37]. MRI is especially valuable for assessing primary OA and subchondral bone in traumatic crater-like lesions, although standard MRI often underestimates the true extent of chondral damage [38,39]. Advanced techniques such as T2 mapping now provide additional insight into collagen matrix integrity and the extent of cartilage injury.

Musculoskeletal ultrasound (MSK-US) is a useful tool for assessing articular cartilage in the knee, offering advantages such as high resolution and accessibility. Normal knee cartilage appears homogeneous and anechoic due to its high water content, with smooth, well-defined margins. Cartilage thickness typically ranges from 1–3 mm, and accurate evaluation depends on proper patient and transducer positioning to obtain an optimal acoustic window. In pathological joints, assessment may be more challenging, as osteophytes can impair visualization through acoustic shadowing [40].

### 1.3. Arthroscopy

Arthroscopy is considered the gold standard for diagnosing intra-articular knee pathology, offering direct visualization of cartilage, menisci, and subchondral bone. It allows precise localization, classification, and measurement of defects, with faster recovery and fewer complications than open surgery [41]. Chondral lesions are evaluated according to the ICRS system, which divides the condyle into nine sectors and grades defect depth from I to IV. Defect height and width are measured with calibrated probes after debridement, as poor-quality margins may compromise graft integration [42]. The choice between an arthroscopic approach and mini-arthrotomy depends on defect size and location; large posterior lesions may require open access.

Recent evidence shows that MRI frequently underestimates cartilage injury severity. In a study by Krakowski et al. (2021), most patients undergoing arthroscopy had multifocal lesions, predominantly involving the femoral condyles and the medial compartment. Comparative evaluation demonstrated that MRI struggles to depict early lesions (ICRS I–II) accurately and often underestimates the extent of deeper lesions (ICRS 3–4). Arthroscopy clearly visualizes softening and superficial defects (grades 1–2), as well as deeper partial-thickness (grade III) and full-thickness defects exposing subchondral bone (grade 4). Although MRI can detect advanced lesions, its resolution remains inferior to direct visualization [43].

The study aimed to evaluate the diagnostic utility of clinical tests and MSK-US in patients with symptomatic focal chondral lesions, using MRI-based ICRS grading as a reference standard for imaging.

Based on these analyses, we sought to construct an integrated clinical algorithm that would allow clinical orientation and risk stratification between isolated chondral lesions (ICRS 3) and more complex osteochondral–mensical lesions (ICRS 4), thus supporting therapeutic decision-making and imaging triage.

Although MRI served as the reference imaging modality for preoperative classification in the present study, all included patients subsequently underwent surgical cartilage repair by mosaicplasty. Intraoperative arthroscopic assessment confirmed the presence and severity of ICRS Grade 3 and Grade 4 focal chondral lesions, supporting the validity of the preoperative MRI-based classification.

## 2. Materials and Methods

### 2.1. Study Design

This observational cross-sectional study included 57 consecutive patients presenting with symptomatic mechanical knee pain and clinically detectable joint effusion. The study was conducted between January 2021 and July 2023 in the Orthopedics and Physiotherapy Departments of the Micromedica Clinic in Piatra Neamț. All examinations and data collection procedures were performed within a single physiotherapy and orthopedic rehabilitation center and adhered to the ethical principles of the Declaration of Helsinki. In order to ensure the replicability of the assessment, it was performed by an orthopedic surgeon who was a member of the team that assessed the patients under the same conditions. The study received approval from the Ethics Committee for Scientific Research of Micromedica Clinics in Piatra Neamț (approval no. 28/21 January 2021). All patients provided written informed consent before participation.

Before inclusion, written informed consent was obtained from each participant. The primary objective was to analyze the diagnostic value of clinical tests and MSK-US in detecting high-grade focal chondral lesions, with MRI used as the reference standard for ICRS grading.

Eligible participants were adults aged between 27 and 50 (40.8 ± 6.8) years who reported load-dependent knee pain, recurrent or persistent swelling, and mechanical limitation of function. All patients were referred for imaging under the suspicion of focal cartilage damage, and only those with MRI-confirmed focal chondral defects were retained for analysis. Patients with KOA, inflammatory joint disease, previous ligament reconstruction, recent fractures, septic arthritis, or any prior prosthetic intervention were excluded to minimize confounding pathology.

All patients included in the study subsequently underwent surgical cartilage repair by mosaicplasty. Intraoperative arthroscopic assessment confirmed the presence and grading of focal chondral lesions (ICRS Grades 3 and 4), thereby validating the preoperative MRI-based classification.

### 2.2. Patient Evaluation

**Figure 1:**
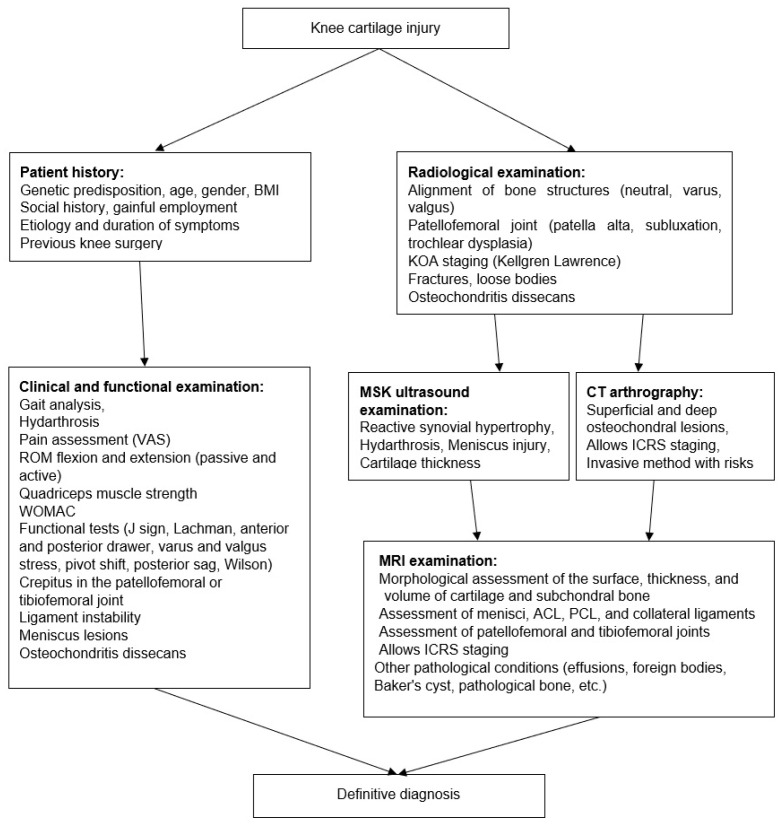
Flow chart of chondral defect assessment through clinical-functional examination and radiological imaging.

Each patient underwent a standardized clinical evaluation conducted by an experienced musculoskeletal clinician. The assessment included a detailed history, quantification of pain using the Visual Analog Scale (VAS), and functional impairment using the Western Ontario and McMaster Universities Osteoarthritis Index (WOMAC). Gait analysis and palpation of the femorotibial joint line were followed by a full ROM assessment, evaluation of patellofemoral crepitus, and inspection for reactive synovial thickening or swelling (Figure 1). Joint effusion was documented using the patellar ballottement and sweeping tests, both of which proved highly sensitive in this cohort.

Functional maneuvers were applied systematically (Figure 1). Wilson’s test was used to provoke medial compartment osteochondral loading, while McMurray’s test was used to evaluate potential meniscal involvement. Additional tests—including the J-sign, Lachman, anterior and posterior drawer tests, pivot-shift test, and varus–valgus stress tests—were performed to assess associated ligamentous or patellofemoral abnormalities. The presence or absence of each clinical sign was recorded as a binary parameter for subsequent statistical analysis.

MSK-US was performed using a high-frequency linear transducer (L64—18 MHz) on a Fujifilm ARIETTA 850 system (Fujifilm Holdings Corporation, Tokyo, Japan) (Figure 1). Scanning was conducted in both longitudinal and transverse planes following standardized protocols for the suprapatellar recess, medial and lateral femorotibial compartments, and the patellofemoral joint. The examination focused on detecting intra-articular effusion, reactive synovial hypertrophy, focal cartilage irregularities, subchondral changes, and meniscal morphology. MSK-US findings were recorded qualitatively as present or absent, and interpreted in conjunction with the clinical profile.

MRI examination was performed using a Siemens Magnetom Aera 1.5 T (Siemens Healthineers, Erlangen, Germany) scanner equipped with a dedicated 16-channel knee coil. The protocol included proton-density-weighted turbo spin-echo (PDw TSE) sequences with and without fat suppression (FS) acquired in axial, coronal, and sagittal planes, as well as T1-weighted turbo spin-echo (T1w TSE) sequences. All chondral lesions were classified according to the ICRS grading system (Figure 1). Grade 3 lesions were defined as deep fissures extending beyond 50% of cartilage thickness, while Grade 4 lesions represented full-thickness cartilage loss with exposed subchondral bone. MRI also served to assess associated pathology such as bone marrow edema, subchondral cysts, meniscal tears, and patellofemoral involvement.

Clinical examination and MSK-US assessments were performed prior to MRI evaluation. Examiners were blinded to MRI findings at the time of assessment. MRI grading was performed by a single experienced musculoskeletal radiologist using ICRS criteria.

Clinical examinations and MSK-US assessments were performed independently and before MRI evaluation. Examiners were blinded to MRI findings at the time of assessment. MRI grading was conducted by a single experienced musculoskeletal radiologist who was blinded to clinical and ultrasound data, to ensure consistency in ICRS classification and reduce inter-observer variability.

## 3. Results

Data preprocessing and statistical analysis were performed using Python libraries (NumPy 2.3.0, Pandas 2.3.3, SciPy, StatsModels). Clinical and MSK-US variables were transformed into binary indicators. Descriptive statistics were derived for all variables. Associations between clinical tests and lesion severity were evaluated using Chi-square tests, while Pearson correlation coefficients were computed to explore relationships between clinical markers and compartment-specific cartilage defects. Odds ratios (OR) and 95% confidence intervals (CI) were calculated using log-transformed methods. Heatmaps and forest plots were generated to visualize correlation patterns and OR distributions. Statistical significance was set at a *p*-value < 0.05.

This methodological framework enabled an integrated clinical and radiological assessment of focal chondral lesions, allowing for the development of a clinically applicable diagnostic algorithm to differentiate between isolated ICRS 3 lesions and more complex ICRS 4 osteochondral injuries.

A total of 57 patients with MRI-confirmed focal chondral lesions of the knee were included in the final analysis. According to MRI-based ICRS grading, 50 patients (87.7%) presented with Grade 3 cartilage defects, while 7 patients (12.3%) were classified as Grade 4. All patients reported mechanical knee pain and exhibited clinical effusion at the time of examination.

Clinically, the most consistent finding across the entire cohort was recurrent joint effusion, present in 100% of cases, regardless of ICRS grade. Pain intensity, measured using the VAS, averaged 5.7 in ICRS 3 lesions and 5.4 in ICRS 4 lesions, indicating a relatively similar symptomatic burden between the two groups.

Functional impairment, quantified using the WOMAC index, showed moderate disability without statistically relevant differences between grades (Table 1). WOMAC total scores indicated a moderate level of functional impairment and were nearly identical between ICRS Grade 3 (64.5) and ICRS Grade 4 (65.7) lesions

Functional clinical tests displayed distinct patterns between ICRS grades. Wilson’s test was the most frequently positive maneuver, being positive in 66% of ICRS 3 cases and in 100% of ICRS 4 cases (Table 2).

In contrast, McMurray’s test was positive in only 30% of patients with ICRS 3 lesions but increased sharply to 86% in ICRS 4 lesions, reflecting frequent meniscal involvement in full-thickness chondral defects.

Instability tests (Lachman, anterior drawer, posterior drawer) were variably positive but did not demonstrate predictive value for chondral severity.

**Table 2:** Frequency of Clinical Test Positivity According to ICRS Grade.

Clinical Test	ICRS 3 (*n* = 50)	ICRS 4 (*n* = 7)
Wilson Test	66%	100%
McMurray Test	30%	86%
J-Test	36%	71%
Lachman Test	52%	86%
Anterior Drawer	moderate	high
Posterior Drawer	0%	0%

Legend: Wilson = most sensitive test for both grades; McMurray positivity was strongly associated with concomitant meniscal pathology, which was significantly more prevalent in ICRS 4 lesions.

Correlation analysis revealed low linear associations between clinical tests and the compartmental distribution of cartilage defects. Pearson coefficients for medial defects ranged between −0.32 and +0.06, while those for lateral defects ranged between −0.06 and +0.32, indicating no significant direct relationship between clinical maneuvers and defect location.

The only statistically significant association identified through Chi-square analysis was observed for McMurray’s test (χ^2^ = 4.29, *p* = 0.038), which demonstrated an inverse relationship with isolated chondral lesions. Negative McMurray responses were more frequent in ICRS 3 lesions, whereas positive McMurray signs were strongly associated with ICRS 4 lesions due to concomitant meniscal pathology (Table 3).

The OR analysis confirmed this pattern. McMurray’s test had a significantly subunitary OR (0.20, 95% CI: 0.05–0.79), indicating that a negative McMurray test strongly predicted isolated focal chondral lesions (ICRS 3).

Wilson’s test showed no significant predictive value (OR 0.74, 95% CI: 0.17–3.15), despite its high clinical sensitivity. Instability tests displayed wide confidence intervals crossing unity, confirming their limited diagnostic relevance in differentiating chondral lesion severity (Table 4).

MRI served as the reference standard for confirmation and grading. Grade 3 lesions were characterized by deep fissures and partial-thickness cartilage loss, while Grade 4 lesions demonstrated full-thickness chondral defects with subchondral bone exposure. MRI additionally identified meniscal damage in 86% of ICRS 4 cases, compared to only 30% of ICRS 3 cases, aligning with clinical observations (Table 5).

Integrating clinical findings with MRI grading allowed for the recognition of distinct diagnostic patterns:

- ICRS 3 lesions were characterized by recurrent effusion, mechanical pain, positive Wilson’s test, and negative McMurray, indicating isolated cartilage pathology.
- ICRS 4 lesions displayed effusion, positive Wilson’s test, and positive McMurray, reflecting combined chondral–meniscal injury.

These results supported the construction of a clinico–radiologic diagnostic algorithm capable of supporting clinical differentiation between isolated chondral lesions and complex osteochondral–meniscal patterns (Table 6).

**Table 6:** Integrated Clinical–Radiologic Pattern for Differentiating ICRS Grades.

Diagnostic Feature	Suggests ICRS 3	Suggests ICRS 4
Mechanical pain	present	present
Recurrent effusion	present	present
Wilson test	frequently positive	always positive
McMurray test	often negative	mostly positive
Meniscal lesions	uncommon (~30%)	very common (~86%)
Subchondral exposure (MRI)	absent	present
Cartilage irregularity (MRI)	deep fissures	full-thickness defect

Legend: This table summarizes the clinical–radiologic decision-making core used in the diagnostic algorithm.

## 4. Discussion

The present study investigated the diagnostic value of clinical examination parameters in identifying high-grade focal chondral lesions of the knee, integrating these findings with MRI-based ICRS staging to construct an evidence-informed diagnostic algorithm. Although the diagnosis of focal cartilage injuries is traditionally reliant on MRI, our findings demonstrate that a structured clinical approach can provide meaningful orientation regarding lesion severity and the likelihood of associated meniscal involvement.

Across the cohort, all patients presented with mechanical knee pain and recurrent effusion, confirming that these symptoms remain universal but nonspecific indicators of intra-articular pathology. WOMAC scores demonstrated moderate functional impairment in both groups, with almost identical mean values (64.5 for ICRS 3 and 65.7 for ICRS 4), suggesting that symptom severity alone does not reliably differentiate between deep partial-thickness and full-thickness cartilage lesions.

Our finding that WOMAC scores did not differ between ICRS Grade 3 and 4 lesions aligns with previous evidence showing that clinical improvement does not necessarily reflect structural cartilage quality [44]. Park et al. (2023) similarly reported significant WOMAC gains despite second-look arthroscopy revealing mainly ICRS 2–3 repair tissue [45]. Lee et al. (2019) also demonstrated that, although defect filling and integration improved after high tibial osteotomy with microfracture, WOMAC outcomes remained comparable between groups [46]. Collectively, these studies support the dissociation between structural cartilage repair and functional recovery, reinforcing that symptomatology alone cannot determine the morphological severity of focal chondral lesions.

Distinct patterns emerged within the clinical tests. Wilson’s test showed high sensitivity across both lesion grades, being positive in two-thirds of ICRS 3 patients and universally positive in ICRS 4 cases. This reinforces the test’s utility in detecting painful overload of the medial femoral condyle, regardless of cartilage depth [47]. Although the Wilson test was initially described for detecting osteochondral pain of the medial femoral condyle, current literature does not demonstrate a correlation between a positive Wilson test and ICRS-based lesion severity [47]. This is consistent with the well-established notion that mechanical pain provocation tests reflect synovial irritation or subchondral overload rather than the morphological depth of a chondral defect.

Although McMurray’s test was the only clinical maneuver showing a statistically significant association, this finding should not be interpreted as reflecting cartilage depth. McMurray positivity primarily indicates concomitant meniscal pathology, which was more prevalent in ICRS Grade 4 lesions. Thus, the observed association reflects lesion complexity rather than intrinsic cartilage severity. Accordingly, McMurray’s test should be viewed as an indirect marker of osteochondral–meniscal involvement rather than a cartilage-specific diagnostic tool.

Statistically, McMurray’s test was the only maneuver with a significant Chi-square association (χ^2^ = 4.29, *p* = 0.038) and exhibited a strong inverse OR for predicting isolated chondral injury (OR = 0.20, 95% CI: 0.05–0.79). These findings underscore its role as a clinical marker capable of distinguishing isolated chondral defects from complex osteochondral–mensical lesions. Given the limited number of ICRS Grade 4 cases, chi-square tests and OR were applied in an exploratory manner to identify association patterns rather than to establish predictive validity. These analyses should be considered hypothesis-generating, and larger cohorts are required to confirm the observed relationships. Although McMurray is not directly connected to cartilage morphology, the biomechanical data reported by Bedi et al. (2010) provide a coherent explanation: large radial meniscal tears substantially increase compartment pressures, leading to chondral overload and progression toward severe cartilage lesions, which in turn increase the likelihood of a positive McMurray test [48].

Correlation analysis further confirmed that conventional clinical tests generally show limited alignment with the anatomical distribution of chondral defects identified on MRI [49,50]. The weak Pearson correlations observed for both medial and lateral lesion patterns indicate that clinical maneuvers lack compartment-specific diagnostic power. This reinforces the concept that while clinical tests may detect functional alterations, they cannot reliably predict the precise location of focal cartilage injury.

MRI served as the reference standard and revealed clear structural differences between grades [50,51]. ICRS 3 lesions presented as deep fissures without subchondral bone exposure, whereas ICRS 4 lesions were full-thickness defects frequently accompanied by bone marrow edema, subchondral exposure, and meniscal tears [51,52]. The high prevalence of associated meniscal pathology in ICRS 4 cases explains the increased positivity of McMurray’s test and the greater frequency of instability-related findings observed in the clinical examination.

A key strength of this study lies in the integration of clinical patterns with MRI confirmation, resulting in the development of a practical, clinician-oriented diagnostic algorithm. This algorithm distinguishes two clinically relevant profiles:

(1) isolated focal chondral lesions (ICRS 3), characterized by mechanical pain, effusion, Wilson positivity, and McMurray negativity;
(2) complex chondral–meniscal lesions (ICRS 4), characterized by combined Wilson and McMurray positivity and supported by MRI evidence of deeper structural compromise. Such pattern recognition can help clinicians stratify patients more efficiently and determine which cases should undergo early MRI evaluation.

While intraoperative arthroscopy confirmed the presence of ICRS Grade 3 and Grade 4 lesions, MRI-based ICRS grading was retained as the analytical reference to ensure standardized, preoperative classification across the cohort. MRI was therefore used as a reference imaging standard for clinical stratification rather than as a definitive staging method, and all findings should be interpreted within this context.

MSK-US provided complementary diagnostic information in this study, particularly in identifying reactive synovial changes, effusion, and indirect signs of cartilage overload. Although MSK-US cannot directly stage cartilage defects with the precision of MRI, its capacity to detect synovial hypertrophy, capsular distension, and irregularities of the chondral contour offers valuable insight into the inflammatory and biomechanical environment surrounding focal lesions [53]. These features were consistently present across both lesion grades, reflecting the mechanical irritation associated with chondral damage rather than its morphological severity.

Current literature supports the utility of MSK-US as an adjunctive tool for early detection of joint effusion, synovitis, and secondary meniscal changes, even when MRI remains necessary for definitive staging [54]. High-frequency linear transducers (10–18 MHz) enable visualization of the superficial cartilage surface and allow dynamic assessment during joint motion. A recent 2025 study reported strong correlations between MSK-US scores and both clinical parameters (VAS r = 0.891; WOMAC r = 0.902) and imaging evaluations (Digital Radiography r = 0.876; MRI r = 0.895) [55]. These findings indicate that MSK-US can reliably reflect both symptomatic burden and structural joint alterations, reinforcing its value as a complementary imaging modality in the diagnostic pathway of focal chondral lesions. Nevertheless, MSK-US remains limited in evaluating deep or posterior cartilage surfaces and cannot accurately determine lesion depth, restricting its ability to serve as a standalone method for ICRS grading.

This study presents several limitations that should be acknowledged. First, the overall sample size was relatively small (*n* = 57), which limits statistical power and may reduce the robustness of subgroup analyses. The small number of patients with ICRS Grade 4 lesions limits statistical power and the stability of inferential statistics. Accordingly, all findings should be interpreted as exploratory and hypothesis-generating rather than confirmatory. Second, the cross-sectional design does not allow evaluation of longitudinal changes or prognostic implications of the diagnostic patterns identified. Third, MSK-US, although valuable for detecting synovitis and effusion, is operator-dependent and unable to reliably assess deep or posterior cartilage surfaces, which may lead to underestimation of lesion depth. Additionally, MRI served as the reference standard without arthroscopic confirmation, potentially affecting the precision of ICRS grading. MRI grading was performed by a single reader, and interobserver agreement was not assessed, which may limit reproducibility. This aspect should be addressed in future studies through multi-reader validation and interobserver reliability analysis. Fourth, although clinical examinations were conducted using a standardized protocol by experienced clinicians, formal interobserver reliability testing (e.g., Kappa statistics) was not performed, which may affect reproducibility. Finally, the single-center setting may limit the generalizability of the results to broader clinical populations.

Potential confounding factors such as physical activity level, symptom duration, and prior conservative treatment were not systematically available for all patients and could not be included in the analysis. These variables may influence clinical symptom severity, cartilage degeneration, and the prevalence of associated meniscal pathology. In addition, the limited number of ICRS Grade 4 cases precluded reliable multivariate regression analysis due to the risk of model overfitting.

Despite these limitations, the integration of MSK-US into the initial diagnostic workflow enhances clinical efficiency. When combined with targeted clinical maneuvers such as Wilson’s test for condylar overload and McMurray’s test for meniscal involvement, MSK-US findings help differentiate inflammatory from mechanical symptoms, identify cases requiring MRI, and facilitate earlier decision-making. This approach aligns with emerging evidence supporting multimodal diagnostic strategies in focal chondral pathology, where MSK-US represents an accessible, cost-effective, and repeatable modality for monitoring synovial responses and guiding clinical suspicion before advanced imaging.

The findings of this study also carry important clinical implications. They support the use of a structured set of clinical maneuvers in the initial assessment of patients with mechanical knee pain and effusion, highlight McMurray’s test as a useful discriminator of full-thickness chondral injury, and demonstrate that functional impairment alone cannot reliably differentiate lesion severity. Overall, this work introduces a pragmatic clinical–radiologic algorithm validated by MRI, the current gold standard, that may improve diagnostic accuracy and enable more timely and appropriate therapeutic decision-making for patients with focal cartilage injuries.

## 5. Conclusions

This study demonstrates that a structured clinical assessment can provide meaningful diagnostic orientation in patients with focal chondral lesions of the knee. Although pain intensity and functional impairment did not differ between ICRS 3 and ICRS 4 lesions, distinct clinical patterns emerged.

Wilson’s test was consistently sensitive for medial compartment overload but did not discriminate between lesion grades. In contrast, McMurray’s test showed significant diagnostic value, strongly correlating with the presence of associated meniscal pathology, which was markedly more frequent in ICRS 4 lesions.

McMurray’s test should be interpreted as a clinical indicator of associated meniscal pathology and lesion complexity rather than a marker of cartilage depth. The proposed algorithm supports clinical orientation and preoperative risk stratification but does not replace definitive arthroscopic assessment.

The findings reinforce the concept that symptomatology and functional scores alone cannot determine the structural severity of focal cartilage injuries. Instead, the combination of targeted clinical maneuvers and MSK-US interpreted alongside MRI offers a reliable strategy for differentiating isolated partial-thickness defects from complex full-thickness osteochondral lesions. This integrated approach may guide more accurate diagnostic decision-making, help prioritize MRI use, and support individualized therapeutic planning.

Future studies with larger cohorts and longitudinal follow-up are warranted to validate the proposed diagnostic algorithm and to explore the prognostic implications of clinical–radiologic patterns in focal chondral disease.

## Figures and Tables

**Table 1:** Baseline Demographic and Clinical Characteristics of the Study Cohort.

Variable	ICRS 3 (*n* = 50)	ICRS 4 (*n* = 7)	Total (*n* = 57)
Age (years, mean ± SD)	*≈44*	*≈46*	*≈45*
VAS Pain Score	5.7	5.4	5.6
WOMAC Total Score	similar	similar	moderate
Mechanical pain present	100%	100%	100%
Recurrent effusion	100%	100%	100%

Legend: All patients had MRI-confirmed focal chondral lesions. Values represent means or percentages.

**Table 3:** Statistical Associations Between Clinical Tests and Medial Chondral Defects.

Clinical Test	Chi-Square (χ^2^)	*p*-Value	Interpretation
Wilson	0.003	0.955	No association
McMurray	4.29	0.038	Inverse association (predictor of isolated ICRS 3)
J-Test	1.20	0.27	Not significant
Lachman	0.02	0.87	Not significant
Anterior Drawer	0.03	0.85	Not significant

Legend: McMurray is the only clinical test with statistical significance.

**Table 4:** Odds Ratios (OR) for Predicting Medial Chondral Defects.

Test	OR	95% CI (Lower–Upper)	Interpretation
Wilson	0.74	0.17–3.15	Non-predictive
McMurray	0.20	0.05–0.79	Negative predictor for isolated lesions
J-Test	0.39	0.10–1.44	Non-significant
Lachman	1.37	0.38–4.90	Non-specific
Anterior Drawer	1.61	0.30–8.53	Non-specific
Posterior Drawer	—	—	Insufficient data

Legend: OR < 1 indicates a higher likelihood of isolated cartilage injury despite a negative test.

**Table 5:** Radiologic (MRI) Features in ICRS 3 vs. ICRS 4 Lesions.

MRI Feature	ICRS 3	ICRS 4
Cartilage thickness loss	deep fissures	full-thickness loss
Subchondral bone exposure	absent	present
Subchondral edema	variable	frequent
Meniscal pathology	30%	86%
Defect location (medial/lateral)	both	both
Bone cysts	rare	more frequent

Legend: MRI served as gold standard for lesion staging.

## Data Availability

The original contributions presented in this study are included in the article. Further inquiries can be directed to the corresponding author.

## Abbreviations

The following abbreviations are used in this manuscript:

ACL	Anterior cruciate ligament
CT	Computed Tomography
KOA	Knee osteoarthritis
LCL	Lateral collateral ligament
MCL	Medial collateral ligament
MRI	Magnetic resonance imaging
MSK-US	Musculoskeletal ultrasound
ICRS	International Cartilage Repair Society
OA	Osteoarthritis
ROM	Range of motion
PCL	Posterior cruciate ligament
VAS	Visual Analog Scale
WOMAC	Western Ontario and McMaster Universities Osteoarthritis Index
